# Fast Renewal of the Distal Colonic Mucus Layers by the Surface Goblet Cells as Measured by *In Vivo* Labeling of Mucin Glycoproteins

**DOI:** 10.1371/journal.pone.0041009

**Published:** 2012-07-17

**Authors:** Malin E. V. Johansson

**Affiliations:** Department of Medical Biochemistry, University of Gothenburg, Gothenburg, Sweden; Emory University School of Medicine, United States of America

## Abstract

The enormous bacterial load and mechanical forces in colon create a special requirement for protection of the epithelium. In the distal colon, this problem is largely solved by separation of the bacteria from the epithelium by a firmly attached inner mucus layer. In addition, an outer mucus layer entraps bacteria to be cleared by distal transport. The mucus layers contain a network of Muc2 mucins as the main structural component. Here, the renewal rate of the inner protective mucus layer was studied as well as the production and secretion of Muc2 mucin in the distal colon. This was performed by intraperitoneal injection of N-azidoacetyl-galactosamine (GalNAz) that was *in vivo* incorporated during biosynthesis of *O*-glycosylated glycoproteins. The only gel-forming mucin produced in the colon is the Muc2 mucin and as it carries numerous *O-*glycans, the granulae of the goblet cells producing Muc2 mucin were intensely stained. The GalNAz-labeled glycoproteins were first observed in the Golgi apparatus of most cells. Goblet cells in the luminal surface epithelium had the fastest biosynthesis of Muc2 and secreted material already three hours after labeling. This secreted GalNAz-labeled Muc2 mucin formed the inner mucus layer. The goblet cells along the crypt epithelium accumulated labeled mucin vesicles for a longer period and secretion of labeled Muc2 mucin was first observed after 6 to 8 h. This study reveals a fast turnover (1 h) of the inner mucus layer in the distal colon mediated by goblet cells of the luminal surface epithelium.

## Introduction

Mucus, the protective cover of all epithelial surfaces, has gel-forming mucins as its major structural component. The mucus layer covering the intestinal epithelium has the MUC2 mucin as the core molecule in this structural network. MUC2 is produced by goblet cells, a cell type specialized for this purpose. The MUC2 mucin is synthesized in the endoplasmic reticulum as a 5,200 amino acid apomucin with cysteine-rich parts in its ends. Dimerization occurs via the C-terminal ends prior to translocation into the Golgi apparatus [1], [2] where the *O*-linked glycans are attached to the abundant serine and threonine residues making up the two central mucin domains. Glycosylation increases the molecular mass of the dimer from about 1 MDa to about 5 MDa. After packing in granulae, the formation of an oligomeric network is completed by N-terminal trimerization [3]. After release and volume expansion, the MUC2 mucin replenishes the luminal mucus. The mucus protects and forms an inner dense mucus layer in the colon that separates the epithelium from the bacteria [4]. On the luminal side of this inner mucus layer, the expanded soluble outer mucus forms the habitat for the colonic bacteria [4], [5]. One general function of the intestinal mucus is to trap microorganisms and to facilitate their distal transport.

Defects in this protective system have been linked to colitis [4], [6], [7] and understanding the intestinal mucus turnover and renewal of the inner protective mucus layer is important for novel ways to improve treatment of inflammatory diseases as ulcerative colitis. To analyze the production, turnover and transport of colonic mucus layers, *in vivo* studies are necessary, studies that have not been performed previously.

Incorporation of azido-sugars during biosynthesis using acetylated GalNAz will label glycosylated molecules. Click chemistry is a fast reaction with such azido-containing molecules using the copper-catalyzed azide-alkyne cycloaddition reaction [8]. This reaction is suitable to attach a fluorescent alkyne to the incorporated GalNAz [9]. This approach can be used to pulse label glycosylated molecules *in vivo.*

Here, I have studied the turnover of glycoconjugates in the distal colon with a focus on the mucus produced by the intestinal goblet cells. *In vivo* labeling of *O-*glycans was performed by intraperitoneal injection of acetylated GalNAz that was incorporated into mucin *O*-glycans by the goblet cells. Labeled mucins were detected using histochemistry with click chemistry in combination with Muc2 immunostaining. Goblet cells of the surface and crypt epithelium were found to display different mucin biosynthetic rates and the inner mucus layer in distal colon was quickly renewed.

## Materials and Methods

### Animals and Ethics Statement

All mice in this study were inbred on the C57Bl/6 background and kept under standardized conditions of temperature (21–22°C) and illumination (12 h light/12 h dark). Animal experimental procedures were approved by the Swedish Laboratory Animal Ethical Committee in Gothenburg (reference number 358-2009) and were conducted in accordance with guidelines of the Swedish National Board for Laboratory Animals.

### Intraperitoneal Injection of GalNAz and BrdU in Mice and Tissue Fixation

The Click iT reagent GalNAz (Invitrogen) was dissolved in 25 µl DMSO and diluted in PBS to a final volume of 500 µl per 2.6 mg reagent for each animal. The solution was supplemented with 3 mg 5-bromo-2'-deoxyuridine (BrdU, Invitrogen) per animal. Injections were given intraperitoneally within the dark period (04:00 a.m.). Three animals per hour were sacrificed for the first 12 h and in addition a 24 h time point was used. The intestine was removed and the colon dissected. Pieces of distal colon containing fecal material were fixed in methanol Carnoy (60% dry methanol, 30% chloroform, 10% glacial acetic acid). Paraffin embedding was preceded with a methanol wash and 4 µm sections were cut and the sections were placed on glass slides.

### Fluorescent Detection of GalNAz in Fixed Tissue and Muc2 Staining

Detection of the azide labeled *O*-glycans were performed by conjugating the azide to a fluorescently labeled alkyne in a copper-catalyzed azide-alkyne cycloaddition reaction [10]. Fixed paraffin embedded sections were dewaxed, hydrated, and washed in PBS. Tissue sections were incubated with 10 µl of reaction mix from the tetramethylrhodamine (TAMRA) glycoprotein detection kit (Invitrogen) and incubated at room temperature for 5 h. After washing in PBS, the samples were blocked in 5% fetal bovine serum and stained with an anti-MUC2 antiserum against a C-terminal peptide (anti-MUC2C3) [4]. Sections (not stained with TAMRA) were subjected to antigen retrieval by boiling in 10 mM citric buffer, pH 6.0 and blocked and incubated with an anti-Muc2 apomucin antiserum, anti-gpA (PH 497) [11]. The specific antibodies were detected with an Alexa488 conjugated anti-rabbit antibody (Invitrogen) and the sections were mounted using ProLong anti-fade mounting medium (Invitrogen). Tissue from animals not injected with GalNAz was used as negative control of the TAMRA alkyne reaction. Two sections from 3 animals per time point were stained and analyzed and representative pictures obtained. The images were acquired using a fluorescence microscope, Eclipse E1000 with a Plan-Fluor 40x/0.75 DIC objective (Nikon) or a confocal microscope, LSM 700 Axio Examiner.Z1 laser scanning confocal microscope, with a Plan-Apochomat 40x/1.3 Oil DIC objective (Zeiss) The pictures were acquired and processed uniformly using the ZEN 2010 software (Zeiss) and Adobe Photoshop.

Quantification of the labeled area of whole luminal surface goblet cells or crypt goblet cell theca was performed on pictures from 3 different regions of the colon sections from 3 labeled mice per time point using the Volocity software (version 6.1, Perkin Elmer). Region of interest (ROI) were determined from Muc2 stained sections to identify longitudinally sectioned surface goblet cells (45 cells per time point). The red stained objects were clipped to ROI excluding non-touching and separate touching with compartmentalization between ROI’s. Individual thresholds were set from background stained areas for each section due to different degree of labeling. The ratio of red area per goblet cell area was calculated and data are presented as mean±SEM. The analysis of the GalNAz-labeled crypt goblet cells was performed accordingly, but the theca area was selected due to mostly cross sectioned cells at this location.

### BrdU Detection by Immunostaining of Fixed Tissue

Fixed paraffin embedded samples were dewaxed, hydrated, and boiled in 10 mM citrate buffer, pH 6.0 for 10 min. The sections were treated with 2M HCl at 37°C for 30 min. The sections were washed in PBS, blocked in 5% fetal bovine serum and stained with anti-BrdU (Sigma) diluted 1/500 in block solution overnight. The BrdU staining was detected by incubation with an anti-mouse-Alexa546 antibody (Invitrogen) and mounted in ProLong anti-fade mounting medium (Invitogen). The images were acquired using a fluorescent microscope, Eclipse E1000 with a 40x/0.75 DIC objective (Nikon).

### Biotin Labeling of Scraped Mucus

Mucus was scraped from 2 cm colon segments adjacent to the distal fixed tissue and samples from mice killed 7, 8 and 9 h after GalNAz-injection was combined (one sample per time point). The mucus was frozen in a small volume of PBS with 2x complete protease inhibitor cocktail (Roche). Thawed mucus was washed for 3 rounds in wash buffer (150 mM NaCl, 50 mM Tris-HCl, 1% triton X-100, pH 7.9) by centrifugation (1,000 x g, 2 min) where the insoluble mucus appears in the pellet fraction. The sample was applied to a Microcon spin filter (MWCO: 100,000 kDa, Millipore) and centrifuged for 10 min at 20,000 x g and washed with 500 µl wash buffer with an additional centrifugation. The filter was inverted and the mucus eluted by a short spin. The mucus was diluted in 50 µl wash buffer and reacted with 50 µl reaction mix of alkyne-biotin (Biotin glycosylation detection kit, Invitrogen) according to manufacturer’s instructions for 24 h at 4°C. The samples were reduced by adding an equal volume of sample buffer with DTT (100 mM final concentration) and incubating at 95°C for 10 min, 37°C for 3 h and 95°C for 10 min.

### Electrophoresis and Western Blot

The reduced sample was separated on an agarose-polyacrylamide composite gel and blotted to PVDF membrane (Millipore) as described previously [12]. The membranes were blocked in 5% milk powder solution and incubated with alkaline phosphatase conjugated streptavidin (1/500, Southern Biotech) or anti-MUC2N3 (1/1000) and anti-MUC2C3 (1/1000) in combination [2], [4] with alkaline phosphatase conjugated anti-rabbit (1/1000, Southern Biotech) as secondary antibody. The blot was developed with NBT-BCIP color reaction (Promega).

## Results

### GalNAz-incorporation in the Distal Colon Epithelium

**Figure 1:**
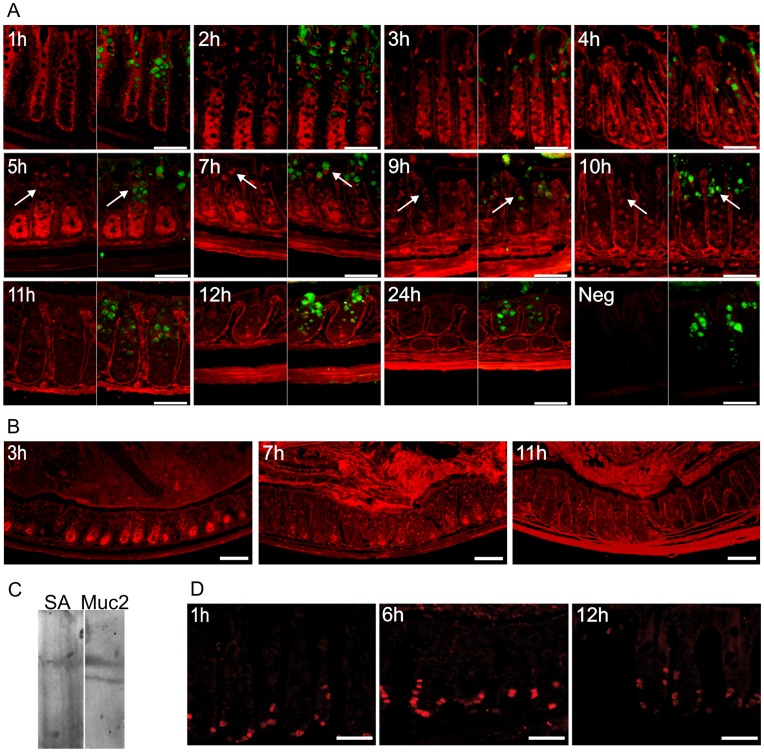
Mucin biosynthesis and cell proliferation of distal colon. A) GalNAz-incorporation during mucin biosynthesis was analyzed every hour for 12 h and at 24 h. Samples from distal colon were fixed after 1 to 24 h, as indicated, and GalNAz was detected by TAMRA (red) in combination with immunostaining of Muc2 (green). GalNAz is first identified in the Golgi region (1 h) and all cells in the crypt bottom are intensely stained during the first 7 h whereafter the staining decreases. Mucus filled goblet cells in the mid to upper crypt are stained green and GalNAz incorporation in the mucin vesicles can be observed from 5 to 10 h of labeling (arrows). The basal membrane and lamina propria are stained after 3 h with highest intensity after 11–12 h and the muscular layers are stained red after 7 h. Tissue not injected with GalNAz but stained with TAMRA was used as a negative control (Neg). Scale bars are 50 µm. B) Low magnification pictures of colon labeled with GalNAz (stained red by TAMRA) for 3, 7 and 11 h. Scale bars are 100 µm. C) Western blot of scraped mucus from mouse colon with GalNAz-labeled mucins separated by agarose-acrylamide electrophoresis. GalNAz was reacted with alkyne-biotin and reduced prior to separation on gel. The blot was detected with streptavidin (SA) or anti-MUC2N3/C3 (Muc2) and the glycosylated Muc2 band is biotinylated demonstrating GalNAz-incorporation into Muc2. D) BrdU was injected simultaneously with GalNAz and was detected by immunostaining (red) after 1, 6 and 12 h. Scale bars are 50 µm.

Distal colon has two mucus layers, where the inner is dense and devoid of bacteria [4]. This inner layer is organized in a stratified manner by the secreted Muc2 mucin. The biosynthesis of mucins was studied by *in vivo* labeling of *O-*linked mucin type glycans by intraperitoneal injection of acetylated GalNAz into mice. The labeling was terminated by sacrificing the mice every hour in a time series of 12 h with an additional time point at 24 h. The GalNAz-labeled molecules were detected in tissue sections of the distal colon with TAMRA using click chemistry in combination with Muc2 immunostaining. The N-acetylgalactosamine (GalNAc) analogue was first, as expected, detected within the Golgi apparatus throughout the crypt where it is being used as a substrate by the peptidyl-GalNAc transferases to produce *O-*linked glycoproteins (Figure 1A, 1 h). As mucins are the most highly *O-*glycosylated proteins and Muc2 is the only gel-forming mucin expressed in the intestine, it will be the most heavily labeled protein in the colon. In the second hour, GalNAz was found in *O*-glycosylated molecules observed as a broader staining within the cells (Figure 1A, 2 h). The goblet cells located in the luminal surface epithelium were also intensely stained after 2 h and this staining could be observed during the following 3 h. Labeled molecules in the lamina propria and the muscular layers were observed after 3 and 7 h respectively, and remained stained thereafter. This indicates a slower turnover of glycosylated extracellular matrix molecules compared to the mucin-type molecules within the epithelium. The goblet cells in the crypts showed an increasing number of stained mucin vesicles from 3 to 10 h and thereafter the number of stained vesicles were fewer, but some still remained stained after 24 h (Figure 1A). Low magnification pictures show staining of tissue and mucus at some selected time point to give an overview of the GalNAz-labeling (Figure 1B). The very intense staining of the cells in the crypt bottom observed during the first 7 h decreased within the next 2 h indicating upward migration and secretion of the labeled proteins. Overall, the observations are in line with incorporation of GalNAz into glycoproteins during the initial hours as a pulse with only limited recycling of the label. Thus these *in vivo* studies follow a cohort of labeled glycoproteins largely made up of highly glycosylated proteins such as the mucins. Labeling was not observed in tissue sections from mice not injected with GalNAz (Figure 1A, Neg). GalNAz-incorporation into the Muc2 mucin was verified by western blot of scraped mucus where GalNAz was reacted with alkyne-biotin and the blots were detected with either streptavidin or MUC2 antiserum (Figure 1C).

Protection of the intestine involves secretion of mucus but also renewal of the epithelial cells. To monitor the cell proliferation, BrdU was injected together with GalNAz. The stained tissue sections revealed that BrdU was incorporated into cells within the bottom of the crypts during the first hour (Figure 1D). These cells had then migrated halfway up the crypts during 12 h, suggesting a turnover time of the epithelial cells in the range of days.

### Mucin Production in the Colonic Goblet Cells located in the Luminal Surface Epithelium

**Figure 2:**
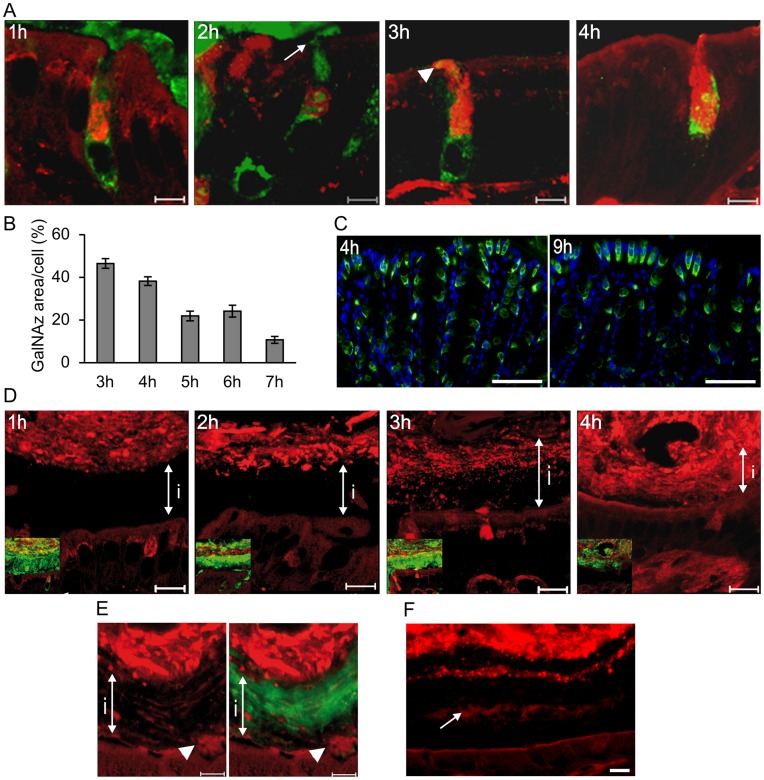
The luminal surface goblet cells secrete mucins that build the inner mucus layer. A) GalNAz-incorporated mucins were detected with TAMRA (red) in the luminal surface epithelium of distal colon. The goblet cells were identified by immunostaining for Muc2 (green). Secreted mucins are not GalNAz-labeled after 2 h (arrow), but are stained by TAMRA 3 h after GalNAz injection (arrowhead). Scale bars are 5 µm. B) Secretion of labeled mucins in the luminal surface goblet cells was determined as the proportion of red stained area of the goblet cells for 3 to 7 h of labeling (mean±SEM). Most labeled mucins were secreted from the cells after 7 h. C) The Muc2 precursor (as found in the endoplasmic reticulum) was observed as the apomucin stained by an antiserum detecting the non-glycosylated Muc2 (green). The nuclei were counterstained by DAPI. The luminal surface goblet cells are stained (apomucin) both at 4 h when secretion of GalNAz-labeled mucins occur and after 9 h when no GalNAz-stained mucins remain in the luminal surface goblet cells indicating continuous renewal of mucins. Scale bar is 50 µm. The inner mucus layer (marked by i) is completely unstained by TAMRA (red) during the first 2 h after GalNAz injection. GalNAz-incorporated mucin containing mucus was secreted after 3 h and this material renewed the inner mucus layer. The inner mucus layer is intensely stained at 4 h and contains almost only GalNAz-labeled mucins. Merged pictures also showing the Muc2 staining (green) of the mucus are displayed in smaller insets. Scale bars are 10 µm. C) The inner mucus layer with Muc2 (green) is still GalNAz-labeled (red) after 9 h but with a lower intensity. The inner mucus layer is indicated with i and newly secreted mucins are marked by arrowheads. Scale bar is 5 µm. D) GalNAz-labeled mucins (red) were in a few instances observed as a line (arrow) within the inner mucus layer. Scale bar is 10 µm.

The surface goblet cells displayed an intense staining of the Golgi apparatus and trans Golgi network (TGN) that resembled a basket-like structure at the first time point analyzed (Figure 2A). Labeled material could be detected in the mucin vesicles containing already formed Muc2 within the next hour, at the same time as labeled mucins were secreted. Mucins secreted before 3 h was not GalNAz-labeled, suggesting a time of 3 h for maturation of the Muc2 mucin from its non-glycosylated form to being secreted. Secretion of labeled mucins continued for a few hours, but the luminal surface cells were not labeled at any later time (Figure 1A). The proportion of the goblet cell area labeled with GalNAz was determined as a measure of mucin production and secretion. After 3 h almost half of the cell area was labeled, but this material was secreted and only 10 % of the area was labeled after 7 h (Figure 2B). The decrease of the stained area reflects secretion of labeled mucins with renewal of unlabeled mucins. Constant renewal of Muc2 was revealed by staining of the non-glycosylated Muc2 precursor as an indicator of new Muc2 translation (Figure 2C). The staining is observed at similar levels both 3 and 9 h after GalNAz injection indicating continuous renewal and secretion of Muc2.

### Turnover of the Inner Mucus Layer in Colon

The mucus secreted from the luminal surface goblet cells replenished the inner mucus layer rapidly by secretion from below. The inner mucus layer was completely unlabeled the first two hours after GalNAz injection as no labeled mucins were secreted (Figure 2B). Secretion of labeled mucins was observed after 3 h indicating that mucin molecules are released starting between 2 and 3 h after GalNAz injection. An area close to the epithelium have mucins secreted from different cells either stained or unstained that is not fully integrated, but during the process of organizing and building the mucus layer the stained and unstained mucin containing mucus is intermixed forming a stratified packed mucus layer. (Figure 2B, 3 h). This staining was less intense, built from both unstained and stained mucins, compared to the completely stained mucus secreted after 4 h. As more of the luminal surface goblet cells secreted labeled mucins, the inner mucus became more evenly and intensely stained (Figure 2B, 4 h). The observation that the mucus layer contained some labeled mucins also at its outer side already after 3 h and that this mucus was replaced by evenly stained mucus after 4 h argues for an inner mucus layer turnover of about an hour. The mucus layer was continuously labeled as mucins secreted from the crypts also contributed to the inner mucus layer, but the staining at 9 h was not as intense as after 4 h (Figure 2C). The inner mucus layer was formed and renewed by secreted and expanded mucins adding layers underneath the already existing mucus. This renewal was in a few instances observed as a stained line within the inner mucus layer reflecting GalNAz-labeled mucins surrounded by unstained secreted mucins (Figure 2D).

### Mucin Vesicles in the Crypt Refill the Goblet Cell Theca

**Figure 3:**
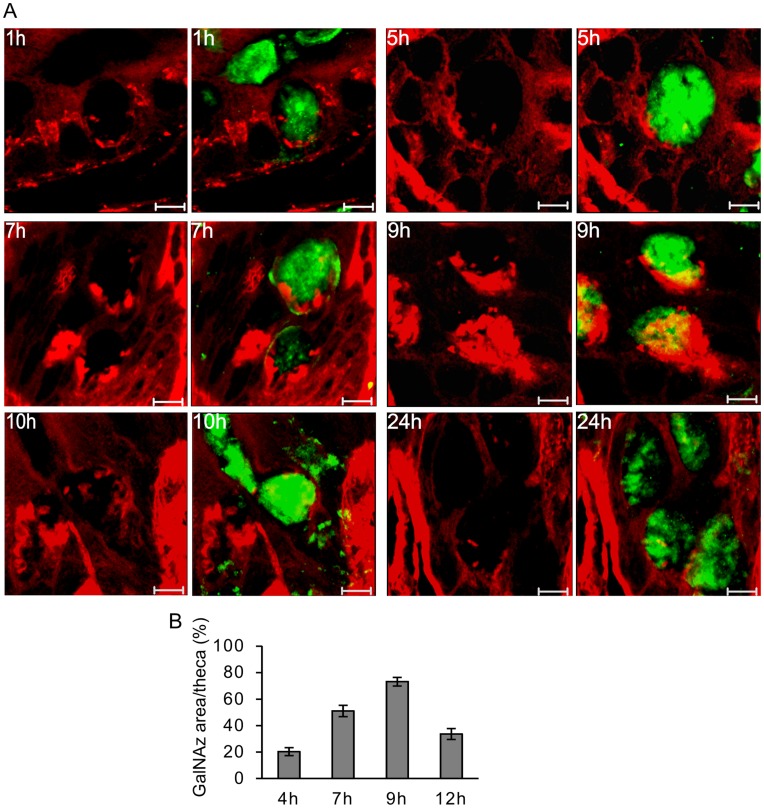
Mucus vesicle formation in goblet cells lining the crypts. A) The goblet cells located in the middle crypt region show initial (1 h) TAMRA staining (red) in the Golgi apparatus where GalNAz was used in the biosynthesis of *O-*linked glycoproteins. Stained mature mucin vesicles can be observed after 5 h and the number of labeled vesicles increases up to 10 h after injection. Most of the labeled vesicles were secreted during 24 h, but a few remained. The TAMRA-stained sections and those combined with the Muc2 staining (green) as merged pictures are shown for all the indicated times. Scale bars are 5 µm. B) The TAMRA stained GlaNAz-containing mucin vesicle area of the theca in the crypt goblet cells is presented for samples labeled for 4, 7, 9 and 12 h. (mean±SEM). Most labeled stored mucins are observed after 9 h.

The goblet cells lining the whole crypt showed Muc2-stained granulae, but the crypt bottom cells had less filled vesicles compared to the upper region of the crypts. The generation of mucin vesicles in the mid-crypt goblet cells was studied during the *in vivo* labeling. The Golgi compartment located at the basal side of the mature Muc2 vesicles was labeled after the first hour, as in the luminal surface epithelium (Figure 3A, 1 h). However, the mature labeled vesicles that emerged from TGN and contributed to the stored mucus vesicle volume did not become labeled until after 4 to 5 hours suggesting a slower biosynthesis compared to what was observed in the luminal surface goblet cells (Figure 3A, 5–7 h). The number of labeled mucin vesicles stored within the crypt goblet cells increased and reached a maximum around 9 to 10 h after GalNAz injection (Figure 3A, 9–10 h). The TAMRA stained area of the goblet cell theca was determined and labeled mucin filled vesicles in the crypt goblet cells peak at 9 h of labeling (Figure 3B).

A few TAMRA-stained vesicles were still visible after 24 h (Figure 3A). These vesicles were located in the center of the theca and could be vesicles that were not secreted with the majority of the granulae. As these were localized in the middle of the stored granulae and not at the periphery as the newly synthesized mucin vesicles, these were probably not formed by new biosynthesis.

### Mucus Secretion from the Goblet Cells Located in the Crypts

**Figure 4:**
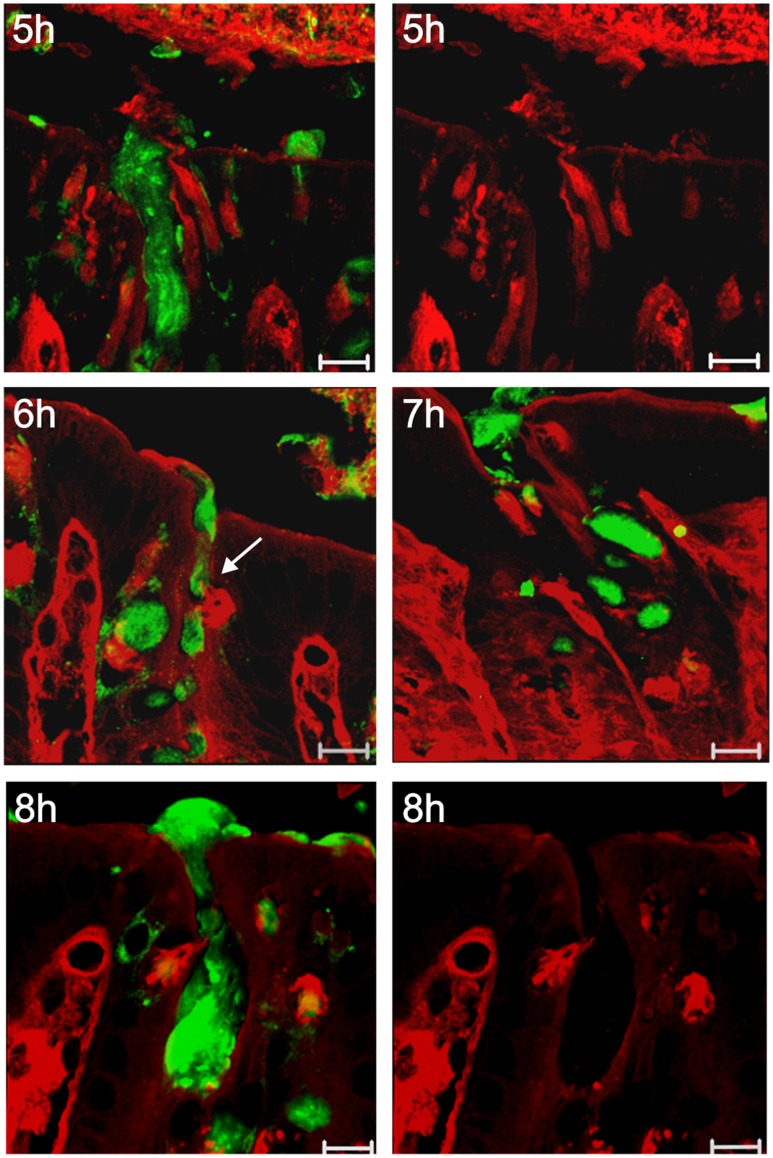
Secretion of GalNAz-labeled mucins from crypt goblet cells. Muc2 (green antibody staining) labeled with GalNAz was detected by TAMRA (red). Within the first 5 h GalNAz-labeled mucin secretion only occurred from the surface epithelium or from cells in the very top of the crypts. Rarely, secretion of labeled mucin was observed after 6 h from goblet cells located in the upper part of the crypt (arrow). In the 8 h samples, TAMRA-staining detected GalNAz-labeled mucin in secreting crypt goblet cells and within the secreted mucus. Scale bars are 10 µm.

GalNAz-labeled mucins stored in the crypt goblet cell vesicles were first observed to be secreted at 6 h by very few cells and secretion was more pronounced from most goblet cells after 8 h (Figure 4). As discussed above, at 5 h only goblet cells of the luminal surface epithelium secreted labeled material that contributed to the inner mucus layer. The crypt goblet cells produced their mucin vesicles over a longer time period (starting after 4–5 h) and secreted these mucins 2–3 h later, a process continued for several hours up to a day.

## Discussion

The distal colon requires an intricate protection system against the high number of bacteria present in the lumen. Combined with the mechanical pressure from propulsion of fecal material, a potentially harmful situation arises where the bacteria are pressed against the epithelial cells. Protection against this danger is largely provided by a compact mucus barrier covering the epithelial cells. This inner mucus layer minimizes mechanical stress on the epithelium and separates it from the bacteria. Preservation of an intact inner mucus layer is important to circumvent unwanted epithelial damage and extensive bacterial exposure. Animals lacking the main mucus component, Muc2, display spontaneous intestinal inflammation [4], [6]. Altered mucus quality caused by mutations in the Muc2 gene or defect *O*-glycosylation also cause colitis [7], [13]. The maintenance of the mucus is a balance of bacterial degradation and distal transport on one hand and renewal by secretion on the other hand. Renewal of the mucus is a key factor in the ability to preserve this protective system. Here, I have determined the turnover of this inner mucus layer to be as fast as 1 h, a surprisingly fast process that emphasizes its importance for protecting the epithelium.

Turnover measurements require *in vivo* labeling such as the method used in this study with injection of acetylated GalNAz and detection of the incorporated azido-sugars by click chemistry. This approach provides an excellent possibility to label and determine the turnover of molecules rich in *O*-glycans, such as mucins, as this GalNAc analogue is equally well utilized by the peptidyl-GalNAc transferases [14]. The large number of *O*-glycans, more than 1,000 per Muc2 monomer unit, permits high incorporation of GalNAz and strong staining of Muc2. There are also some limitations with the technique as the click reaction with TAMRA interferes with the subsequent Muc2 immunostaining resulting in limited co-staining of the GalNAz-labeled tissue sections. Fixation with Carnoy’s fixative is a good way to preserve the mucus, but the treatment during the click reaction easily damages the mucus and complicates the analysis.

The mucin and mucus layer turnover is considerably faster than the cell renewal of the epithelium. The proliferative cells labeled at injection, illustrated by BrdU incorporation, only reached the lower parts of the crypt after 12 h. The BrdU-labeling suggest a turnover of all the colon epithelial cells of more than 2 days. The goblet cells have been proposed to have an even slower renewal rate than the enterocytes in colon [15]. The mucin turnover in colon is thus much faster than the cell renewal and each goblet cell must renew its stored mucin vesicles more than once.

Secretion of labeled mucins was first observed after 3 to 4 h from the luminal surface goblet cells. This is in accordance with results from studies using radioactive labeled sugars [16], [17]. GalNAz-labeled mucins were first (after 2 h) detected in the cup-shaped Golgi apparatus and TGN. *In vivo* labeling of the Golgi apparatus using radioactively labeled sugars has previously been determined to occur within 30 min [17]. During the next hour, GalNAz was observed in mature Muc2 vesicles and labeled mucins were also secreted from luminal surface goblet cells. This reveals a fast incorporation and emptying of GalNAz-labeled mucins without any subsequent accumulation of mucin vesicles. After 6 h, almost no surface goblet cells were stained indicating a complete renewal of the mucin vesicles in these cells during this time.

In the crypt goblet cells, GalNAz-stained vesicles were observed after 5 h and the number of these vesicles increased with time. The vesicles emerged from the cup-shaped Golgi and TGN compartments before appearing at the outer rim of the theca. Later on, GalNAz-labeled mucins were observed to fill most of the closely packed theca vesicles in the goblet cells.

The continuous renewal of the inner mucus layer was the result from secretion by the luminal surface goblet cells, but mucus secretion from the top of the crypts also contributed. The renewal was set as the time from unstained to stained mucin containing mucus and was estimated to occur within one hour. The newly secreted GalNAz-labeled mucins were observed as a compact and intensely stained material, but when it expanded to form the inner mucus layer the GalNAz intensity decreased. This reflects the expansion of mucus after secretion and that it spreads out under the inner stratified mucus layer to continuously build up the mucus in an organized way [4], [5]. Stained material observed as a band within the inner mucus layer argues for such a model for mucus layer renewal. The inner mucus is then converted to a more voluminous loose outer mucus layer at the outer surface of the inner mucus layer. The loose mucus is mixed with the luminal material which was strongly stained in all the GalNAz-injected samples and the conversion from the inner to the outer mucus layer could thus not be analyzed. This background staining could be due to unspecific fluorescence from undigested food, unspecific staining of the bacteria, or GalNAz utilized by bacteria in the intestinal lumen.

Mucin secretion from the crypt goblet cells at the top of the crypt was first observed after 6 h and was frequent after 8 h. Secretion from the crypts contribute to the inner mucus layer and protect the crypt epithelium. The large amount of mucus stored in the crypt goblet cells is a depot of mucus that can be released by stimulation.

These results show that goblet cells at the surface and crypt express, store and secrete mucins differently. Different responses to stimulatory agents have also been reported for luminal surface and crypt located goblet cells [18], [19]. Goblet cells at the surface epithelium in the colon show a fast mucin synthesis and do not store mucin vesicles long enough to form the typical goblet cell shape as observed in the crypts. The crypt goblet cells produce mucins throughout the whole crypt but larger amounts of mucins are observed in the cells of the upper part.

In the intestine, the mucin synthesis and secretion is rapid in the distal region which coincides with a large bacterial load. The importance of bacterial exposure to produce a functional mucus barrier is demonstrated by germ-free animals where the inner mucus layer is very thin [4], but can be restored by exposure to bacterial components [20]. The mucus layer maintenance is also known to be stimulated by bacterial fermentation products [21].

In conclusion, the mucus production and secretion is a continuously ongoing process with a renewal of the inner protective mucus in the distal colon within an hour. A fast renewal of the mucus barrier prevents microbial contact with the epithelial cells. Defects in renewal and formation of the inner mucus layer could allow bacteria to reach the epithelium and have implications for the causes of colitis.

## References

[1] Lidell ME, Johansson MEV, Mörgelin M, Asker N, Gum JR (2003). The recombinant C-terminus of the human MUC2 mucin forms dimers in CHO cells and heterodimers with full-length MUC2 in LS 174T cells.. Biochem J.

[2] Asker N, Axelsson MAB, Olofsson SO, Hansson GC (1998). Dimerization of the human MUC2 mucin in the endoplasmic reticulum is followed by a N-glycosylation-dependent transfer of the mono- and dimers to the Golgi apparatus.. J Biol Chem.

[3] Godl K, Johansson MEV, Karlsson H, Morgelin M, Lidell ME (2002). The N-termini of the MUC2 mucin form trimers that are held together within a trypsin-resistant core fragment.. J Biol Chem.

[4] Johansson ME, Phillipson M, Petersson J, Velcich A, Holm L, et al. 2008) The inner of the two Muc2 mucin-dependent mucus layers in colon is devoid of bacteria.. Proc Natl Acad Sci U S A.

[5] Johansson ME, Holmen Larsson JM, Hansson GC (2010). Microbes and Health Sackler Colloquium: The two mucus layers of colon are organized by the MUC2 mucin, whereas the outer layer is a legislator of host-microbial interactions. Proc Natl Acad Sci U S A .. 1006451107 [pii];10.1073/pnas.1006451107 [doi].

[6] Van der SM, de Koning BA, De Bruijn AC, Velcich A, Meijerink JP (2006). Muc2-deficient mice spontaneously develop colitis, indicating that MUC2 is critical for colonic protection.. Gastroenterology.

[7] Heazlewood CK, Cook MC, Eri R, Price GR, Tauro SB (2008). Aberrant mucin assembly in mice causes endoplasmic reticulum stress and spontaneous inflammation resembling ulcerative colitis.. PLoS Med.

[8] Tornoe CW, Christensen C, Meldal M (2002). Peptidotriazoles on solid phase: [1,2,3]-triazoles by regiospecific copper(i)-catalyzed 1,3-dipolar cycloadditions of terminal alkynes to azides. J Org Chem 67: 3057–3064.. jo011148j [pii].

[9] Laughlin ST, Bertozzi CR (2009). Imaging the glycome.. Proc Natl Acad Sci U S A.

[10] Agard NJ, Baskin JM, Prescher JA, Lo A, Bertozzi CR (2006). A comparative study of bioorthogonal reactions with azides.. ACS Chem Biol.

[11] Hansson GC, Baeckstrom D, Carlstedt I, Klinga-Levan K (1994). Molecular cloning of a cDNA coding for a region of an apoprotein from the 'insoluble' mucin complex of rat small intestine.. Biochem Biophys Res Commun.

[12] Schulz BL, Packer NH, Karlsson NG (2002). Small-scale analysis of O-linked oligosaccharides from glycoproteins and mucins separated by gel electrophoresis.. Anal Chem.

[13] Fu J, Wei B, Wen T, Johansson ME, Liu X (2011). Loss of intestinal core 1-derived O-glycans causes spontaneous colitis in mice. J Clin Invest 121: 1657–1666.. 45538 [pii];10.1172/JCI45538 [doi].

[14] Hang HC, Yu C, Kato DL, Bertozzi CR (2003). A metabolic labeling approach toward proteomic analysis of mucin-type O-linked glycosylation.. Proc Natl Acad Sci U S A.

[15] Grant TD, Specian RD (1998). Proliferation of goblet cells and vacuolated cells in the rabbit distal colon. Anat Rec 252: 41-48. 10.1002/(SICI)1097-0185(199809)252:1≶41::AID-AR5>3.0.CO;2-H [pii]..

[16] Neutra M, Leblond CP (1966). Synthesis of the carbohydrate of mucus in the golgi complex as shown by electron microscope radioautography of goblet cells from rats injected with glucose-H3.. J Cell Biol.

[17] Neutra M, Leblond CP (1966). Radioautographic comparison of the uptake of galactose-H and glucose-H3 in the golgi region of various cells secreting glycoproteins or mucopolysaccharides.. J Cell Biol.

[18] Neutra MR, O'Malley LJ, Specian RD (1982). Regulation of intestinal goblet cell secretion. II. A survey of potential secretagogues.. Am J Physiol.

[19] Phillips TE, Phillips TH, Neutra MR (1984). Regulation of intestinal goblet cell secretion. III. Isolated intestinal epithelium.. Am J Physiol.

[20] Petersson J, Schreiber O, Hansson GC, Gendler SJ, Velcich A (2011). Importance and regulation of the colonic mucus barrier in a mouse model of colitis. Am J Physiol Gastrointest Liver Physiol 300: G327–G333.. ajpgi.00422.2010 [pii];10.1152/ajpgi.00422.2010 [doi].

[21] Gaudier E, Rival M, Buisine MP, Robineau I, Hoebler C (2009). Butyrate enemas upregulate Muc genes expression but decrease adherent mucus thickness in mice colon. Physiol Res 58: 111–119.. 1271 [pii].

